# Perioperative Myocardial Injury after Adult Heart Transplant: Determinants and Prognostic Value

**DOI:** 10.1371/journal.pone.0120813

**Published:** 2015-05-05

**Authors:** Luca Salvatore De Santo, Michele Torella, Gianpaolo Romano, Ciro Maiello, Marianna Buonocore, Ciro Bancone, Alessandro Della Corte, Nicola Galdieri, Gianantonio Nappi, Cristiano Amarelli

**Affiliations:** 1 Chair of Cardiac Surgery, Department of Surgical and Medical Sciences, University of Foggia, Foggia, Italy—Casa di Cura Montevergine (AV); 2 Department of Cardiovascular Surgery and Transplants, V. Monaldi Hospital, Naples, Italy; 3 Department of Cardiothoracic Sciences, Second University of Naples, Naples, Italy; University of Catania, ITALY

## Abstract

**Background and Aim of the Study:**

Implications of Cardiac troponin (cTnI) release after cardiac transplantation are still unclear. This study disclosed risk factors and prognostic implication of cTnI early levels in a single centre cohort operated on between January 1999 and December 2010.

**Methods:**

Data on 362 consecutive recipients (mean age: 47.8±13.7, 20.2% female, 18.2% diabetics, 22.1% with previous cardiac operations, 27.6% hospitalized, 84.9±29.4 ml/min preoperative glomerular filtration rate) were analyzed using multivariable logistic regression modeling. Target outcomes were determinants of troponin release, early graft failure (EGF), acute kidney injury (AKI) and operative death.

**Results:**

Mean cTnI release measured 24 hours after transplant was 10.9±11.6 μg/L. Overall hospital mortality was 10.8%, EGF 10.5%, and AKI was 12.2%. cTnI release>10 μg/L proved an independent predictor of EGF (OR 2.2; 95% CI, 1.06–4.6) and AKI (OR 1.031; 95% CI, 1.001-1.064). EGF, in turn, proved a determinant of hospital mortality. Risk factors for cTnI>10 μg/L release were: status 2B (OR 0.35; 95% CI, 0.18-0.69, protective), duration of the ischemic period (OR 1.006; 95% CI, 1.001-1.011), previous cardiac operation (OR 2.9; 95% CI, 1.67-5.0), and left ventricular hypertrophy (OR 3.3; 95% CI, 1.9-5.6).

**Conclusions:**

Myocardial enzyme leakage clearly emerged as an epiphenomenon of more complicated clinical course. The complex interplay between surgical procedure features, graft characteristics and recipient end-organ function highlights cTnI release as a risk marker of graft failure and acute kidney injury. The search for optimal myocardial preservation is still an issue.

## Introduction

Troponin release after cardiac surgery, first described in 1991, is detectable virtually in every patient. Given the inherent high sensitivity and specificity for myocardial injury, the release of such biomarkers is a valuable tool to estimate both the burden of the surgical procedure and the underlying vulnerability of the myocardium. The evidence from a recent authoritative meta-analysis and several publications is that, despite a variety of confounding factors prevents definitive conclusion about effect size and cut-off value, such a release is a predictor of both early and midterm survival [1–3]. Unlike in non-transplantation settings, data regarding risk factors and consequences of troponin release after cardiac transplantation are less clear. It has been associated with donor characteristics, effectiveness of myocardial protection and length of ischemic time [4–6] and data are accumulating that demonstrate a relation with increased early postoperative morbidity and mortality [7]. Recent analysis by our institution highlighted such a prognostic pattern [8, 9]. Aim of the present retrospective observational study was to examine the predictors and prognostic implications of troponin release early after heart transplant surgery.

## Materials and Methods

### Study Setting and Patient Sample

Study setting was the Department of Cardiothoracic and Respiratory Sciences of the Second University of Naples located in an affiliated teaching hospital (V. Monaldi Hospital). As described earlier by these authors, at this institution, patients’ information is collected on a daily basis using standardized case report forms for relevant clinical peri-operative variables. Data are entered into a computerized database including 100 variables, which was programmed to accept only data falling within pre-specified ranges. All queries are resolved by referring to the patients’ original records [10]. Data concerning the specific transplant process of care are prospectively collected in the Universal Transplant Database, which has similar standards of accuracy [11]. These two databases were merged for the purpose of the present analysis. Three hundred sixty-two consecutive patients undergoing cardiac transplantation between January 1999 and December 2010, constituted the study sample.

### Study Design and Aims

This retrospective single center cohort study aimed to determine predictors and clinical implications of troponin I release early after orthotopic heart transplantation (HTx).

### Surgical and Critical Perioperative Care

Donor-recipient matching was mainly based on ABO blood type compatibility and size. Size matching generally did not exceed 20% of body weight as per center policy, favoring moderate oversizing in those recipients displaying severe pulmonary artery hypertension. Prospective human leukocyte antigen (HLA) matching was deemed mandatory for recipients with high levels (>20%) of panel reactive anti- HLA antibodies. A retrospective HLA matching was obtained in almost all the other cases. Graft harvesting was allowed in younger donors (males < 40 years of age and females < 45) when there was no evidence of preexisting heart disease and impaired myocardial dysfunction at echocardiography. When considering acceptance of older heart donors, beside thorough evaluation of past medical history and risk factors, every effort was made to obtain a coronary artery angiography to rule out atherosclerotic lesions. Particular care was taken in evaluating echocardiographic data on left ventricular hypertrophy (LVH) of the donor organ. A case by case thorough evaluation implied, on the one hand, the overall technical quality of the ultrasound examination and the eventual execution of a second scan and, on the other hand, the congruity of these evidences with hemodynamic parameters, ECG findings (Sokolow-Lyon and Cornell criteria), inotropic support and donor history. As described earlier by these authors, high inotropic support of donor heart, defined as dopamine or dobutamine at a dose of 10 μg/kg/min or as the need for norepinephrine, did not prevent graft usage when there was echocardiographic evidence of LVEF >40% after haemodynamic optimization. Need for excessive inotropic support (dopamine or dobutamine at a dose of 20 μg/kg/min or similar doses of other adrenergic agents despite optimization of preload and after load) was instead considered a contraindication to organ harvesting as per current guidelines [8–10]. Other details on donor suitability criteria are reported elsewhere [8–10]. Relevant surgical steps were cold ischemic storage after donor heart perfusion with Celsior cardioplegic solution, standard biatrial anastomotic technique and peroperative graft surface cooling with saline in order to improve myocardial protection. Principles of postoperative care and protocols of immunosuppressive care have been fully elucidated elsewhere [8, 9]. Overall study design and inherent standards of care comply with the principles of both the 2000 Declaration of Helsinki and the 2008 Declaration of Istanbul. The study has been thoroughly reviewed by the Ethics Committee (V.Monaldi Hospital), which waived the need for informed consent.

### Baseline Data and Clinical Outcomes Definitions

LVH of donor organ was graded according to the recommendations by the American Society of Echocardiography: the greater of two measurements recorded in the Database (interventicular septum and posterior wall thickness) was used to classify the allograft as having no LVH (< 1.1cm), mild LVH (1.2–1.3 cm) and moderate-severe LVH (>1.4cm) [13].

Early graft failure (EGF), as reported elsewhere, was defined as a mono- or biventricular low output syndrome with a cardiac index < 2 l/min/m^2^, higher filling pressures (right atrial pressure or pulmonary capillary pressure > 20 mmHg) in the first 24 h with the need of high inotropic support (dobutamine dose > 10 γ/kg/min and/or isoprenaline > 0.05 γ/kg/min or need for adrenaline or levosimendan), systemic (nitroprusside, nitroglycerin) and/or pulmonary vasodilators (PGE2, NO and sildenafil), intra-aortic balloon pump (IABP), prolonged intubation with high inhaled oxygen concentrations [8, 9]. Patients experiencing low CO syndrome for technical reasons (two cases of pulmonary artery stenosis and one case of narrow left atrial cuff) were excluded from the analysis. Definition of multiple organ failure (MOF) conforms with guidelines and is reported elsewhere [9]. Incidence of acute kidney injury (AKI) according to RIFLE criteria was investigated through the changes in plasma creatinine concentration as defined by the difference between baseline concentration and the highest concentration during the stay in ICU. The gender modulated Modification of Diet in Renal Disease equation was used to estimate glomerular filtration rates (GFR) [14]. The classification for acute kidney injury by the Acute Dialysis Quality Initiative Workgroup was constructed [15]. Δ GFR > 50% was the target study event. Fatality (hospital mortality) was defined as all deaths occurring during the hospital period in which the operation was performed and those deaths occurring after hospital discharge but within 30 days of the procedure. As per protocol the Troponin I samples analyzed in this study were those drawn at 24 hours after the end of the surgical procedure in all cases. Such time point was chosen on the basis of previous analyses of these authors and by extensive review of the limited available literature on this topic [4–9].

### Statistical Analysis

Data were summarized using standard statistical descriptors such as means, standard deviations, frequencies and percentages. Univariate parametric or non-parametric tests (chi-square Pearson test, unpaired student *t* test, Mann-Whitney test) were appropriately executed in order to address clinical and statistical relevance of variables on the primary end points. Variables that were not linearly related were also mathematically transformed, categorized along appropriate cut points or converted into multiple dichotomous variables. The logistical fit of each continuous variable was examined and dichotomization was performed by choosing, as a cutoff, the value that indicated an endpoint risk greater than the median risk of the overall population. Factors with values of P ≤ 0.10 on univariate tests were included in the multivariable models. Multiple logistic regression was conducted using the forward stepwise method. Since the series included patients operated on over a decade, changes in the pattern of perioperative care may have occurred. To account for such changes date of operation was forced into the multivariable models. All statistical analyses were performed with SPSS 10.0 (SPSS Inc, Chicago, Ill).

## Results

### Study samples features

**Fig 1:**
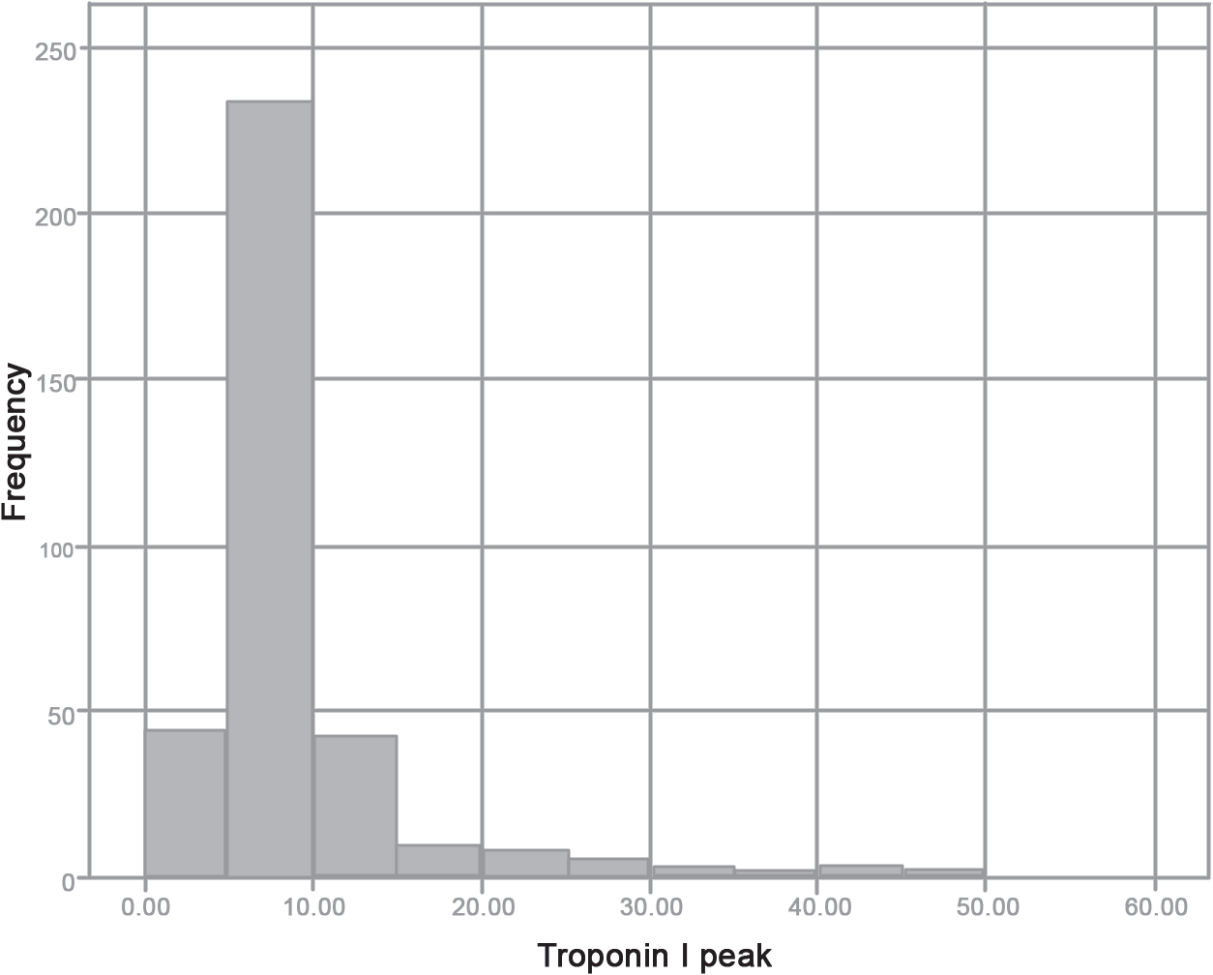
Overall pattern of 24-hour troponin release.

**Fig 2:**
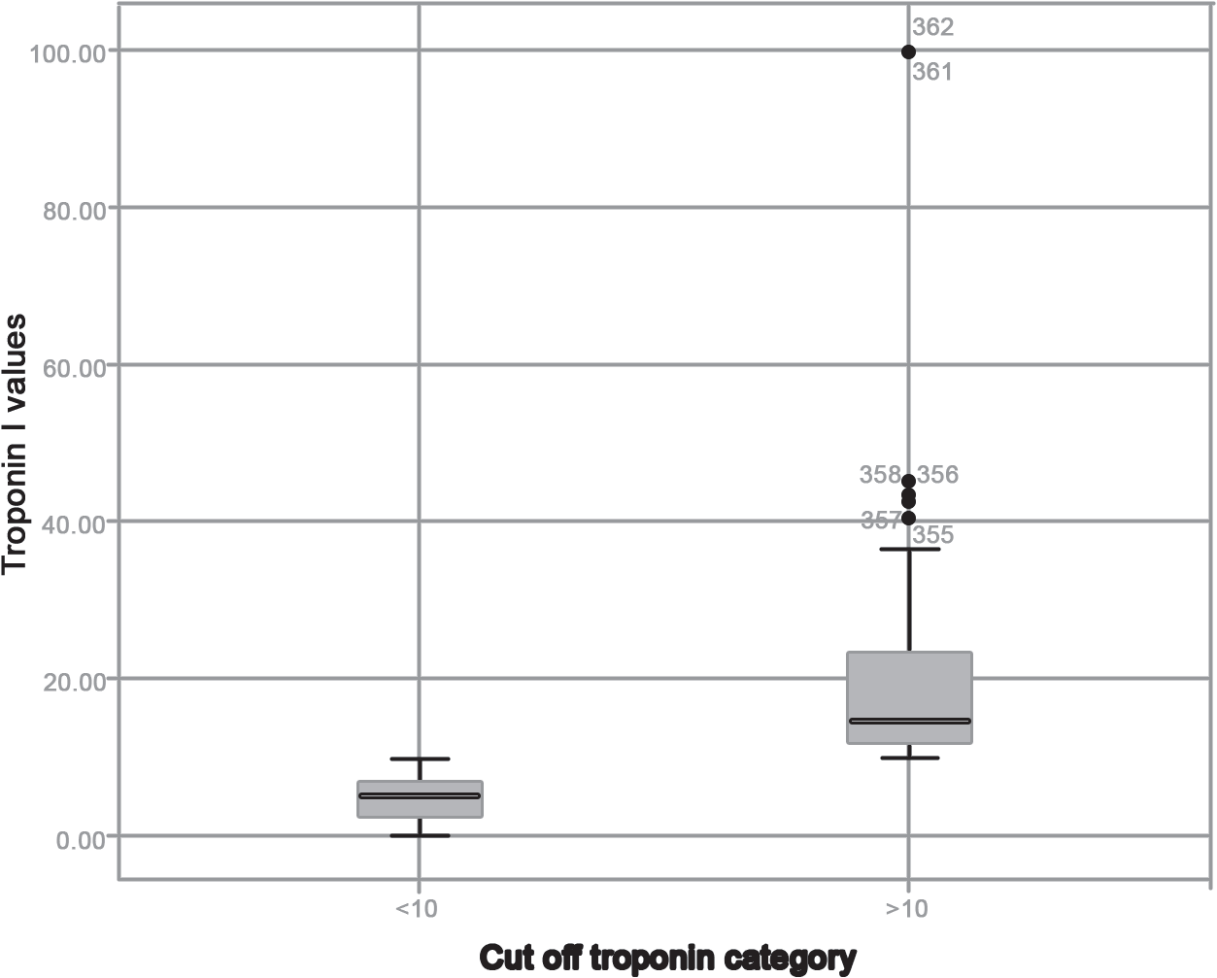
Pattern of distribution of troponin release stratified according to the cut-off.

Tables 1 and 2 summarize main recipients, donors and transplants characteristics. Table 3 reports post transplant outcomes including troponin release, incidence of AKI, acute graft failure and in-hospital death. Mean troponin I release was 10.9±11.6 μg/L. Overall pattern of troponin release is reported in Fig 1. Eighty-three patients (22.9% of the pts) had a troponin release >10 μg/L. Fig 2 shows patient distribution according to troponin release. Outcomes of patients with early troponin release > 10μg/L were significantly poorer (i.e. incidence of EGF: 20.5% vs 7.5%; incidence AKI: 26.5% vs 7.9%; hospital mortality rates: 20.5% vs 7.9%)

**Table 1:** Recipients characteristics.

	N = 362 pts
**Waiting time (months)**	8±10
**Age (mean ± SD)**	47.8±13.7
**Female sex (%)**	73(20.2%)
**BSA (kg/m** ^**2**^ **)**	2.5±10.7
**PVRI (Wood Units)**	4.1±2.6
**Indications Coronary Artery Disease**	142 (39.2%)
**Indications Myopathy**	138 (38.1%)
**Indications Valvular Heart Disease**	23(6.4%)
**Indications Miscellaneous**	59(16.3%)
**Previous cardiac surgery**	80 (22.1%)
**Preoperative eGFR (mean ± SD; ml/min)**	84.9±29.4
**Pre-operative Hemoglobin (mean ± SD; g/dL)**	13±2.2
**Diabetes (%)**	66 (18.2%)
**Hospitalized at time of transplant (%)**	100(27.6%)
**United Network for Organ Sharing Status 1**	53(14.6%)
**United Network for Organ Sharing Status 2A**	48(13.3%)
**United Network for Organ Sharing Status 2B**	261(72.1%)

BSA: body surface area; eGFR: estimated glomerular filtration rate; PVRI: pulmonary vascular resistance index.

**Table 2:** Donor and transplant characteristics.

	N = 362
**Age (mean ± SD)**	32.7±12.8
**Female sex (%)**	134 (37%)
**D/R size matching <0.8 (%)**	58(16%)
**High inotropic support**	111 (30.7%)
**Donor cause of death (%) head trauma**	217(59.9%)
**Donor cause of death (%) stroke**	135(37.3%)
**Donor cause of death (%) other**	10(2.8%)
**Donor LVH (%) No**	192 (53%)
**Donor LVH (%) Mild**	145(40%)
**Donor LVH (%) Moderate-severe**	25 (7%)
**Graft ischemic time (minutes: mean ± SD)**	180.4±48

D/R: donor/recipient; LVH: left ventricular hypertrophy

**Table 3:** Outcomes.

Outcomes	
**Total units of blood (mean ± SD)**	2.8 ± 4.6
**Graft Failure (%)**	38 (10.5%)
**Troponin I > 10μg/liter**	83 (22.9%)
**MOF (%)**	35 (9.6%)
**Peak eGFR (ml/min/1.73m** ^**2**^ **)**	69.1 ± 25.4
**AKI (eGFR > 50% or CVVH)**	44 (12.2%)
**ICU length of stay (days; mean ± SD)**	13 ± 14
**In-hospital length of stay(days; mean ± SD)**	25 ± 19
**Hospital mortality(%)**	39 (10.8%)

AKI: acute kidney injury; eGFR: estimated glomerular filtration rate; ICU: intensive care unit; MOF. Multiorgan failure

### Multivariable Regression modeling for EGF, AKI and fatality

Univariate predictors of graft failure were: graft ischemic time, donor/recipient size matching <0.8, previous cardiac operation, troponin I release > 10μg/L, hospitalization at time of transplant. Univariate predictors of AKI were: previous cardiac surgery, mechanical circulatory support, ischemic time, extracorporeal circulation (ECC) length, troponin I release > 10μg/l, and transfusion of > 4 blood units. Univariate predictors for hospital mortality were: non-idiopathic cardiomyopathy, previous cardiac operation, hospitalization at time of transplant, donor age, early graft failure, AKI, troponin I release > 10μg/L and blood transfusions. Multivariable logistic regression analysis for such outcomes is summarized in Table 4. Troponin release > 10μg/l proved an independent predictors of EGF. EGF in turn was a prognosticator of hospital mortality.

**Table 4:** Logistic regression modelling for graft failure, AKI, and Hospital Mortality.

**Graft failure**	**β**	**OR**	**IC 95%**	***p***
**Troponin I > 10μg/liter**	0.8	2.2	1.06–4.6	0.034
**D/R size matching <0.8**	0.9	2.6	1.2–5.6	0.017
**Previous cardiac operation**	1.3	3.8	1.8–7.8	<0.0001
**AKI (> 50% decrease in eGFR /CVVH)**	**β**	**OR**	**IC 95%**	***p***
**Graft ischemic time**	0.08	1.008	1.011–1.16	0.025
**Troponin I > 10μg/liter**	0.30	1.031	1.001–1.064	0.022
**Transfusion > 4 blood units**	0.082	1.08	1.011–1.16	0.024
**Previous cardiac operation**	0.854	2.35	1.11–4.9	0.025
**Hospital Mortality**	**β**	**OR**	**IC 95%**	***p***
**Blood transfusion**	0.14	1.15	1.06–1.24	<0.0001
**Previous cardiac operation**	1.4	4.1	1.71–9.6	0.001
**Graft failure**	2.4	11	4.4–27.2	<0.0001

AKI: acute kidney injury; D/R: donor/recipien

### Multivariable Regression modeling for troponin release

Univariate predictors of cTnI > 10μg/L release were: status at transplantation, donor gender, PVRI, length of ischemic time, previous cardiac operation, donor/recipient (D/R) size matching < 0.8 and left ventricular hypertrophy. Independent predictors for such a myocardial enzyme leakage are reported in Table 5.

**Table 5:** Logistic regression modelling for troponin release >10μg/l.

	β	OR	IC 95%	*p*
**Status 2B**	-1.05	0.35	0.18–0.69	0.002
**Graft ischemic time**	0.006	1.006	1.001–1.011	0.032
**Previous cardiac operation**	1.06	2.9	1.67–5.0	<0.0001
**Donor LVH > 1.4cm**	1.2	3.3	1.9–5.6	<0.0001

LVH: left ventricular hypertrophy

### Comment

This study depicts the largest group of adult cardiac transplantation patients who have had cTnI levels correlated with perioperative morbidity and mortality reported so far. Rates of hospital mortality, incidence of graft failure as well as that of acute kidney injury, the two most dreaded early post-transplant complications, are consistent with contemporary reports. The pattern of troponin release observed in this series is similar to that reported by Fiocchi and Minami [5, 16]. TnI release is a recognized biochemical marker of perioperative preservation effectiveness [4, 6]. It is important to emphasize that increased susceptibility to humoral and cellular rejection as well as to cytomegalovirus infections and chronic graft vasculopathy are known correlates of acute ischemic damage in the transplant setting [17]. Effectiveness of preservation results from a complex interplay between type of cardioplegic solution, surgical procedure, and quality of the graft itself. Indeed, graft left ventricular hypertrophy, older age, lack of pre-harvesting coronary angiograms and long distance procurement all individually and, even more, synergistically imply an intrinsic higher vulnerability. Given the critical donor shortage such a pattern deserve maximal consideration. There is consistent evidence that the longer the graft ischemia the lower the chances of early and late survival. Indeed longer ischemic time implies a significant increase in the incidence of primary graft failure, need for mechanical support and renal replacement therapy [18, 19]. Such a vicious circle is mirrored by the findings of this study: a TnI elevation > 10μg/L is predicted by longer ischemic time and emerged as a risk factor for EGF and AKI. This pattern reinforces newly generated evidence that optimization of perioperative hemodynamic performance is the mainstay for effective renal preservation strategy in high-risk surgical settings and expands previous knowledge of heart transplant series [7–9, 20].

A recent report from the United Kingdom Cardiothoracic Transplant Audit has newly brought attention on the importance of both cold and warm ischemia for survival after heart transplant [21]. In the present analysis a previous cardiac operation emerged as a predictor of elevated troponin I release which might be indeed a surrogate of intraoperative ischemic damage. Topical cooling during graft implant, as adopted in this experience, though simple and easy, might be less effective than controlled reperfusion strategies in high risk procedures [5, 22]. Up to now, no consensus has been reached as to which of the available cardioplegic solutions and additives provides the best myocardial protection [23]. In the present series all the allografts were protected by means of Celsior solution and early survival data are consistent with both those previously reported by the present authors and those forwarded by multi-institutional database analyses [12, 24]. Donor-recipient matching is a key issue for transplant outcomes [25–26]. Donor-recipient size matching < 0.8 in this series proved a predictor of graft failure adding to current knowledge [25]. Interestingly recipient status 2B at transplantation proved protective as to troponin release. These findings are certainly a surrogate of the most accurate donor/recipient matching in the setting of non-emergent transplantation. Finally, left ventricular hypertrophy of the donor heart has been long believed to increase the risk of ischemic graft injury so portending higher mortality. Limited and inconsistent data regarding these risks are available and this might explain the wide variability in the pattern of usage of such donors between individual transplant centers. Pattern of LVH in the donor population mirrors those reported in large contemporary series [27]. In the present analysis moderate-severe LVH proved an independent predictor of elevated troponin release mirroring the results forwarded by Wever Pinzon et al. and Kuppahally and coworkers and does suggest a judicious usage of such donors [26, 28].

### Study overview

Several study limitations should be considered for a thorough data interpretation. First the single centre setting, though guarantee of a uniform process of care, closely reflects the influence of specific standards of clinical practice and a unique patient population that may have led to results not readily transferable to other patient populations. On the other side, correctness of statistical tools in balancing for confounders make results of this analysis, in an unselected consecutive series of patients experiencing almost all clinically relevant events, transferable. Second, the observational design intrinsically prevents definition of causality, allowing just the disclosure of association between clinical factors. Third, all the biases inherent to a retrospective design apply. Fourth, the series includes patients operated on during nearly a decade and patterns of referral and perioperative care may have changed. Nevertheless timing of surgery never showed up as an outcome predictor (see paragraphs on statistical analysis and results). Fifth, the small number of target events may influence information regarding results of survival analyses. However, the determinants disclosed are perfectly in accordance with those reported in the literature. Sixth, the echocardiographic measurements of wall thickness were performed by cardiologists at different donor facilities and not by a single operator. Besides such pattern prevented the calculation of LV mass which requires cubing of several primary measurements. Finally, reporting of troponin I using assays from different manufacturers may prevent direct comparisons of data from individual institutions and prevent the ultimate definition of cutoff values for target events. Nevertheless, the pattern of myocardial enzyme release reported in this series is consistent with those reported in contemporary experiences and predisposing factors as well as clinical correlates are in accordance with current knowledge.

## Conclusions

Present study significantly adds to current knowledge on this topic, and, to the best of our knowledge, this is the largest series so far that evaluated the prognostic implications of cTnI release after heart transplant [29–33]. Moreover, it is the first to focus broadly on both morbidity and mortality as well as on the determinants of early troponin release. Indeed, most of published reports focused on the correlation of later troponin release with rejection and subsequent survival [30, 32–33]. Other studies tried to correlate the pattern of release of these biomarkers with newly introduced markers of ischemic-reperfusion injury in small prospective series [29]. In the present study, myocardial enzyme leakage clearly emerged as an epiphenomenon of more complicated clinical course. Indeed, the complex interplay between surgical procedure features, graft characteristics and recipient end-organ function highlights cTnI release as a powerful marker of risk, bearing in mind that statistical tools are intended to disclose clinical meaningful factors association and not direct causation. Several surgical factors as well as donor features and practice patterns proved independent predictors of myocardial enzyme leakage. Knowledge of such factors may enhance preoperative risk stratification and allow management modification. In this respect, the findings of this study reinforce those forwarded by the literature and portends for a reconsideration of current allocation protocols and a judicious evaluation of distant heart grafts and those with altered left ventricular morphology. Implementation of cold ischemic storage and intraoperative reperfusion strategies should increasingly confront with the benefits of the developing technique of ex vivo machine perfusion. The cost-effectiveness of alternative surgical strategies to best support patients aggravating on the waiting list should be thoroughly evaluated whenever in need for an urgent transplantation. Finally, given the inherent prognostic implications, this study suggests that measurement of troponin levels should be factored in directing management of patients even after interventions such as cardiac transplantation.
